# *Undaria pinnatifida* extract attenuates combined allergic rhinitis and asthma syndrome by the modulation of epithelial cell dysfunction and oxidative stress: *Undaria pinnatifida* attenuates CARAS

**DOI:** 10.3724/abbs.2024190

**Published:** 2024-12-24

**Authors:** Zhen Nan Yu, Yan Jing Fan, Thi Van Nguyen, Chun Hua Piao, Byung-Hoo Lee, So-Young Lee, Hee Soon Shin, Tae-Geum Kim, Chang Ho Song, Ok Hee Chai

**Affiliations:** 1 Department of Anatomy Jeonbuk National University Medical School Jeonju 54896 Republic of Korea; 2 School of Medicine Liaocheng University Liaocheng 252000 China; 3 Department of Pulmonary and Critical Care Medicine Yantai Yuhuangding Hospital Yantai 264000 China; 4 Department of Food Science and Biotechnology Gachon University Seongnam 13120 Republic of Korea; 5 Division of Food Functionality Research Korea Food Research Institute Wanju 55365 Republic of Korea; 6 Food Biotechnology Program Korea University of Science and Technology Daejeon 305-350 Republic of Korea; 7 Department of Bio-Convergence Science Jeongup Campus of Jeonbuk National University Jeongup 56212 Republic of Korea; 8 Institute for Medical Sciences Jeonbuk National University Jeonju 54896 Republic of Korea

**Keywords:** combined allergic rhinitis and asthma syndrome, *Undaria pinnatifida*, nasal epithelial barrier dysfunction, antioxidant, IL-33

## Abstract

*Undaria pinnatifida* ( *U*. *pinnatifida*) has long been a part of the human diet and medicine. Although *U*. *pinnatifida* has been reported to have immunomodulatory, anti-inflammatory, anti-diabetic and antibacterial activities, its specific effect on patients with combined allergic rhinitis and asthma syndrome (CARAS) has not been clarified. In this study, the anti-allergic and anti-inflammatory effects of *U*. *pinnatifida* extract (UPE) are investigated in a mouse model of ovalbumin (OVA)-induced CARAS. The oral administration of UPE inhibits allergic responses by reducing OVA-specific immunoglobulin levels. As a result, the symptoms of early reactions are also improved. UPE inhibits the accumulation of inflammatory cells and attenuates the expression of Th2 cytokines in both nasal and bronchoalveolar lavage fluid. Furthermore, UPE treatment inhibits the NF-κB/MAPK signaling pathway in lung homogenates. Additionally, UPE prevents shedding of the nasal mucosal epithelium, protects the integrity of the epithelium, enhances the expression of E-cadherin at the junction of epithelial cells, and inhibits the degradation of ZO-1 and occludin in the airway epithelium. In addition, UPE ameliorates dysfunction of the nasal epithelial barrier by enhancing antioxidant properties and downregulating the expression of the inflammatory factor IL-33. These results suggest that UPE may treat CARAS by modulating epithelial cell dysfunction and oxidative stress.

## Introduction

Combined allergic rhinitis and asthma syndrome (CARAS) is a new form of uniform airway inflammation composed of upper respiratory tract inflammation, ″allergic rhinitis″, and lower respiratory tract inflammation, asthma″ [1]. The majority of patients typically have both allergic asthma and rhinitis. According to epidemiological data, up to 40% of people with asthma also have allergic rhinitis, and up to 80% of people with asthma have nasal symptoms [2]. This finding indicates a close relationship between the two symptoms. Therefore, the term CARAS has been used to characterize these two diseases as one disease because their origin, etiology, pathophysiology and shared treatment are closely related [ 1, 3] .

Both allergic rhinitis and asthma involve a type 2 immune response (Th2 spectrum) and are inflammatory diseases characterized by the infiltration of eosinophils, T cells, and mast cells; the release of multiple mediators, chemokines, and cytokines; and the synthesis of systemic IgE [4]. The main transcription factor, nuclear factor-kappa B (NF-κB), is responsible for triggering the inflammatory response in CARAS [5]. In addition, mitogen-activated protein kinases (MAPKs) regulate Th2 cytokine expression and influence the differentiation of inflammatory cells, including eosinophils and Th2 cells [6].

Previous reports have shown that reactive oxygen species (ROS) is also involved in the pathogenesis of asthma, mediating changes in protein and lipid oxidation that lead to pathological changes in respiratory epithelial cells, increased vascular permeability, barrier dysfunction, smooth muscle contraction, mucus overproduction, or airway hyper-responsiveness [7]. The barrier function of the airway epithelium depends on tight and adherent junctions formed by intercellular interactions of proteins such as zonula occludens-1 (ZO-1), epithelial cadherin, and occludin [8]. The function of these proteins is to protect the underlying tissue from harmful and allergenic stimuli [9]. Damage to tight junctions is an important cause of epithelial barrier breakdown during lung or nasal inflammation [10]. Interleukin 33 (IL-33) is defined as an endogenous ″danger″ signal or ″alarm″ released by epithelial cells after cell and tissue damage during trauma or inflammation to alert the immune system. The nuclear cytokine IL-33 is derived from tissues and belongs to the IL-1 family, which is abundantly expressed in epithelial cells and fibroblast-like cells [11]. IL-33 and suppression of tumorigenicity 2 (ST2)/IL1RL1 (IL-33 receptor) play a role in allergen-induced eosinophil inflammation, mucus epithelial chemotaxis, and airway hyperresponsiveness and promote Th2 polarization to participate in the immune response [12].

*Undaria pinnatifida* ( *U*. *pinnatifida*), a brown alga commonly grown in the oceans of China, Korea and Japan, has long been a part of the human diet and medicine. *U*. *pinnatifida* is well known for its phenolic compounds [13] with antioxidant [14], anti-inflammatory [15], anti-diabetic [16], anti-proliferative [17] and antibacterial [18] activities. The galactan component of *U*. *pinnatifida* is mainly composed of galactose-fucoidan (galactofucans), which has anti-inflammatory and antitumor properties [19]. Moreover, fucoidan-derived galactose has a significant antiviral effect on human cytomegalovirus, herpes simplex virus type 1 (HSV-1), and HSV-2 [20].

Antihistamines, anticholinergics, intranasal corticosteroids, and oral anti-leukotrienes are options for treating allergic rhinitis and asthma [21]. However, these treatments can cause side effects such as drug resistance. Therefore, we analyzed the therapeutic potential of *U*. *pinnatifida* in the CARAS experimental model.

## Materials and Methods

### Animals

All experiments were performed using adult male BALB/c mice (6 weeks old) purchased from Damool Sciences (Daejeon, Korea). The mice were bred in the laboratory for 1 week under controlled conditions of 23–25°C, a relative humidity of 10%–50%, and a light/dark cycle of 12/12 h each. All animal experiments were performed in compliance with the animal care and use protocols of the Jeonbuk National University Laboratory Animal Center (JBNU 2021--0115).

### Preparation for UPEs

Dried *U*. *pinnatifida* was obtained from Gijang, South Korea. The *U*. *pinnatifida* was extracted using 70% ethanol at 50°C for 6 h, concentrated (at 50°C using a rotary evaporator BÜCHI Labortechnik AG, Flawil, Switzerland), and freeze-dried (freeze dryer OPERON; Gimpo, South Korea). The obtained powder was stored at –80°C. We used UPLC-Q-TOF MS to examine the different components. Owing to the high sugar content of *U*. *pinnatifida*, it is necessary to determine the sugar content before using UPLC-Q-TOF MS and then remove the sugar content. We expect polysaccharides and phytochemicals to be the functional components of UPEs. The results demonstrated the polysaccharide analysis ( Supplementary Figure S1B). When we compared the molecular sizes before and after dialysis, we discovered that after dialysis, large molecules, mostly between 63,000 and 160,000 Da, were present, whereas before dialysis, molecules less than 6300 Da in size were more common ( Supplementary Figure S1A). Furthermore, a comparison of sugars before and after dialysis revealed an increase in the amount of galactose, confirming that galactose is a functional component of this UPE ( Supplementary Figure S1B). For CARAS mouse model therapy, UPE powder was dissolved in normal saline at concentrations of 50, 100 and 200 mg/kg body weight. Dexamethasone (Dex): 2 mg per kg of body weight was used as a positive control group for comparison with the UPE treatment groups.

### CARAS murine model establishment and UPE treatment

As described previously [22], the mice were randomly assigned to six groups ( *n*=6): the control group, OVA group,UPE (50, 100 and 200 mg/kg) groups, and Dex (2 mg/kg) group. Briefly, on days 1, 8 and 15, OVA-induced CARAS model mice were sensitized via an intraperitoneal injection of 200 μL of saline containing 50 μg of OVA (grade V; Sigma, St Louis, USA) bound to 1 mg of aluminum hydroxide (Thermo Scientific, Rockford, USA). One week after the last sensitization, on day 21 to day 23, the OVA, Dex, and UPE groups were challenged by inhaling ultrasonically nebulized 5% OVA solution in saline for 20 min. The control group received saline alone. On day 24 to day 30, the mice in the OVA, UPE, and Dex treatment groups received an intranasal challenge in each nasal cavity with 20 μL of 10 mg/mL OVA solution. The mice were given UPE and Dex orally 1 h before intranasal challenge with OVA once a day from day 15 to day 30. The mice in the OVA group and control group were given a sham volume of saline. The mice were sacrificed 24 h after the last OVA challenge on day 31 ( Supplementary Figure S2).

### Observation of nasal symptoms

The mice were subjected to intranasal administration of 10 mg/mL OVA in a volume of 20 μL per nasal cavity for 7 consecutive days, starting on day 25 and ending on day 31. Following the previous challenge, we captured the sneezing and rubbing actions on video for a duration of 15 min. Observers, unaware of the study′s purpose, subsequently quantified these actions.

### Nasal lavage fluid (NALF), bronchoalveolar lavage fluid (BALF) collection and cell counting

After the mice were sacrificed, we incised the trachea to collect BALF. We introduced 1 mL of ice-cold saline into the lungs twice and extracted it each time via a tracheal cannula. One milliliter of sterile saline was gently pumped into the nasal cavity through an 18-gauge catheter to obtain NALF. NALF and BALF were collected following centrifugation at 2236 *g* for 10 min at 4°C. The supernatant was transferred to another clean tube and kept at –80°C for subsequent analysis of cytokine levels. We resuspended the cell pellets in cold sterile saline and used a hemocytometer to determine the total number of cells. To determine differential cell counts, 150 μL of NALF or BALF was centrifuged onto slides. A cell cytospin apparatus (Centrifuge 5403; Eppendorf, Hamburg, Germany) was used at a temperature of 4 degrees Celsius for 10 min at a speed of 1000 revolutions per minute. The relative populations of different cell types were assessed via a Diff-Quik staining kit (1-5-1 Wakinohama-Kaigandori; Chuo-Ku, Kobe, Japan). Inflammatory cells were counted under a light microscope (Nikon Instruments Inc, Melville, USA) at a magnification of 400×.

### Histopathological analysis of lung and nasal tissues

After the BALF and NALF were collected, the heads and lungs of the mice were removed and preserved in 10% formalin for three days. The samples were dehydrated with ethyl alcohol and xylene. The tissues were fixed in paraffin and cut into 4 μm slices. The sections were stained with hematoxylin and eosin (H&E) (Sigma) to observe their general morphology and with periodic acid-Schiff (PAS) (Sigma) to assess goblet cell proliferation.

### Assessment of serum OVA-specific immunoglobulin levels

We collected blood from the orbital venous plexus of anesthetized mice 24 h after the final OVA challenge. The samples were subsequently centrifuged (2236 *g*/min, 10 min, 4°C) to separate the serum and stored at –80°C until analysis. ELISAs were performed on serum samples to detect OVA-specific IgE (BioLegend, San Diego, USA), OVA-specific IgG1 (Cayman, Ann Arbor, USA), and OVA-specific IgG2a (Ehondrex, Redmond, USA) according to the manufacturers’ instructions.

### ELISA

The supernatant was collected from NALF and BALF and used to quantify the release of cytokines. The levels of the Th1 cytokines IFN-γ (R&D Systems, Minneapolis, USA) and IL-12 (R&D Systems), the Th2 cytokines IL-4 (R&D Systems), IL-5 (R&D Systems), and IL-13 (CUSABIO, Houston, USA), and the levels of NF-κB and p-NF-κB (Cell Signaling Technology, Beverly, USA) in the NALF and BALF were quantified using respective cytokine assay kits. Cytokine quantitation kits (Mybiosource, San Diego, USA) were used to measure oxidative stress mediators, including nuclear factor-like 2 (Nrf-2), heme oxygenase-1 (HO-1), superoxide dismutase (SOD) and malondialdehyde (MDA), as well as the tight junction-associated proteins ZO-1 and occludin, according to the manufacturer’s protocols.

### Immunohistochemistry examination

For E-cadherin and ST2 immunohistochemistry, a rabbit-specific HRP/DAB (ABC) kit (Abcam, Cambridge, USA) and anti-E-cadherin and ST2 antibodies (Abcam) were used. In brief, the sections were heated for 15 min at 100°C in 1× citrate buffer (Abcam), pH 6.0. After they were blocked for 10 min in hydrogen peroxide, they were subjected to two 10-min washes with PBS. We incubated the samples for 30 min at room temperature for protein blocking to prevent non-specific background staining. After the slides were incubated with an anti-E-cadherin primary antibody (1:2000; Cell Signaling Technology) or an anti-ST2 primary antibody (1:500; Cell Signaling Technology) overnight at 4°C in a humidified box, they were rinsed three times with PBS for five minutes each. Following the application of biotinylated goat anti-polyvalent antibodies for 90 min at room temperature, the samples were rinsed three times with PBS for 5 min each, and then, streptavidin-peroxidase was added for 10 min. The samples were then dehydrated in ethanol and xylene, stained with 3,3′-diaminobenzidine, and counterstained with hematoxylin. Finally, we mounted the slides on stands and scrutinized them under a ×400 magnification optical microscope (Nikon Instruments Inc).

### Western blot analysis

The lung tissues were homogenized and lysed. After that, the tissue homogenate was centrifuged (5 min, 4°C, 2236 *g*), and the supernatants were collected as the total protein extracts. The bicinchoninic acid technique was used to determine the protein concentrations. Protein was separated via 10% sodium dodecyl sulfate-polyacrylamide gel electrophoresis and then transferred to a polyvinylidene difluoride membrane. After the membrane was blocked with 5% dry milk for one hour at room temperature, it was incubated with a β-actin monoclonal antibody (Cell Signaling Technology) and a MAPK antibody (Cell Signaling Technology) overnight at 4°C with gentle shaking. The secondary antibody (1:1000) coupled with bovine serum albumin (GenDEPOT) was incubated with the membrane for 90 min at room temperature. The ECL reagents identified immunoreactive bands. Bio-Rad Laboratories′ Image Lab Version 4.0 software was used to quantify the band intensities. The internal control for protein loading was β-actin.

### Statistical analysis

The data were analysed via Graph Pad Prism software (v 8.0; La Jolla, San Diego, USA). The data are expressed as mean ± SEM. Tukey’s test was used after one-way ANOVA of the group differences. *P*<0.05 was considered statistically significant.

## Results

### UPE ameliorates nasal symptoms in OVA-induced CARAS mice

**Figure 1:**
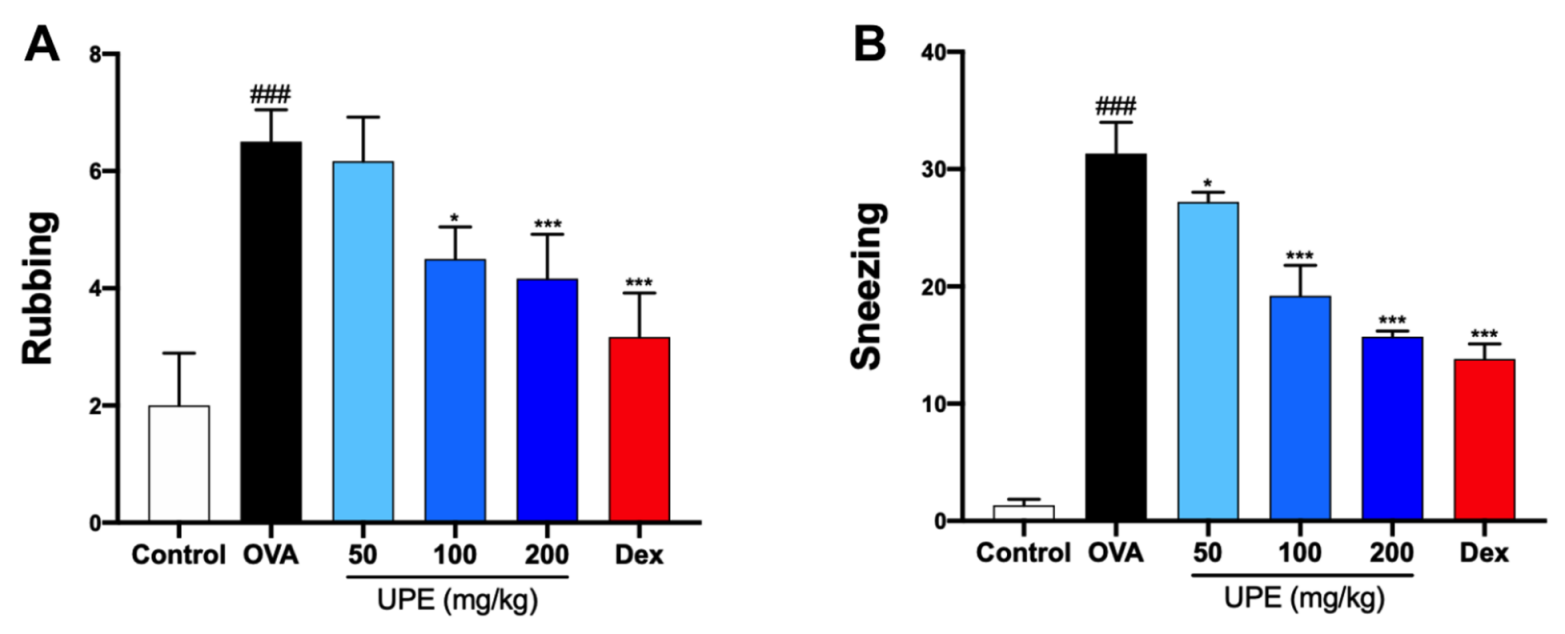
Figure 1 Reduction in allergic nasal symptoms caused by UPE in OVA-induced CARAS mice (A) Rubbing and (B) nasal sneezing were evaluated for 15 min after the last OVA challenge. Data were presented as the mean ± SEM of triplicate experiments. One-way ANOVA was used to compare the differences among multiple groups. *P < 0.05, ***P < 0.001 vs OVA group; ### P < 0.001 vs control group. n = 6.

In this study, OVA sensitization and challenge were used to establish a mouse model of CARAS. To determine the effect of UPE on early allergic symptoms, the frequency of rubbing and sneezing was determined 15 min after the last OVA intranasal challenge. Compared with the control group, the OVA group rubbed and sneezed much more frequently ( Figure 1A,B). The UPE and Dex groups sneezed and rubbed significantly less than the OVA group did.

### UPE reduces the number of inflammatory cells in the NALF and BALF of OVA-induced CARAS mice

**Figure 2:**
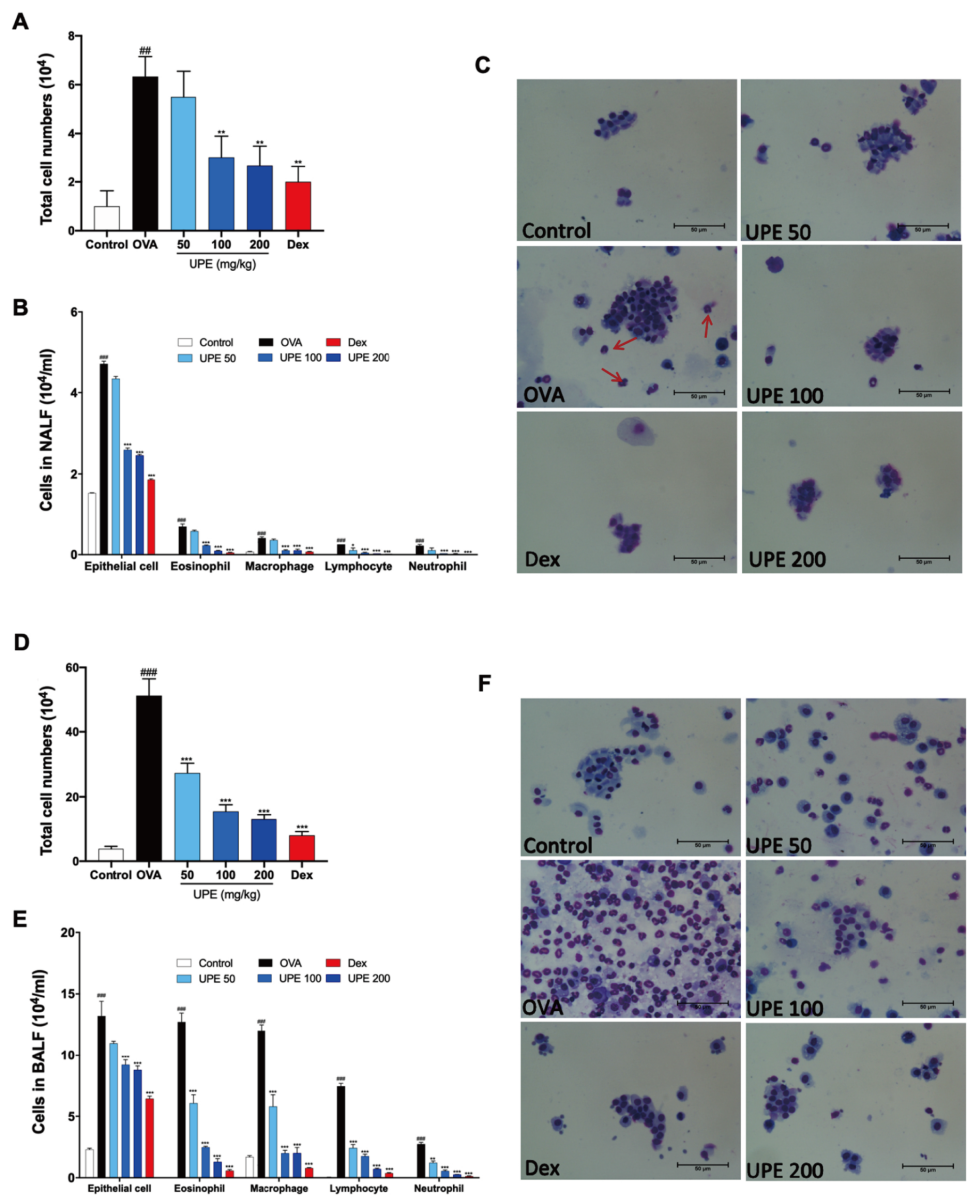
Figure 2 Reduction in the number of inflammatory cells in the NALF and BALF caused by UPE in OVA-induced CARAS mice (A,D) Total number of cells in the NALF and BALF. (B,E) Differential cells were isolated via cytospin. (C,F) Cytospin preparation was stained with Diff-Quik. The red arrows indicate eosinophils. Data were presented as the mean ± SEM of triplicate experiments. One-way ANOVA was used to compare the differences among multiple groups. *P < 0.05, ***P < 0.001 vs OVA group; ### P < 0.001 vs control group. n = 6. Scale bars: 50 μm.

After the last OVA challenge, NALF and BALF samples were collected, and the total cell count was determined. Compared with those in the control group, the total cell count and inflammatory cell count were notably elevated in the NALF and BALF of the OVA group. However, after UPE or Dex treatment, the total cell count and inflammatory cell count were decreased ( Figure 2A,D). Compared with the control group, the OVA group had more inflammatory cells, such as epithelial cells, eosinophils, neutrophils, lymphocytes, and macrophages. However, after treatment with UPE or Dex, the number of these inflammatory cells was clearly reduced ( Figure 2B, C,E,F).

### UPE alleviates airway inflammation in OVA-induced CARAS mice

**Figure 3:**
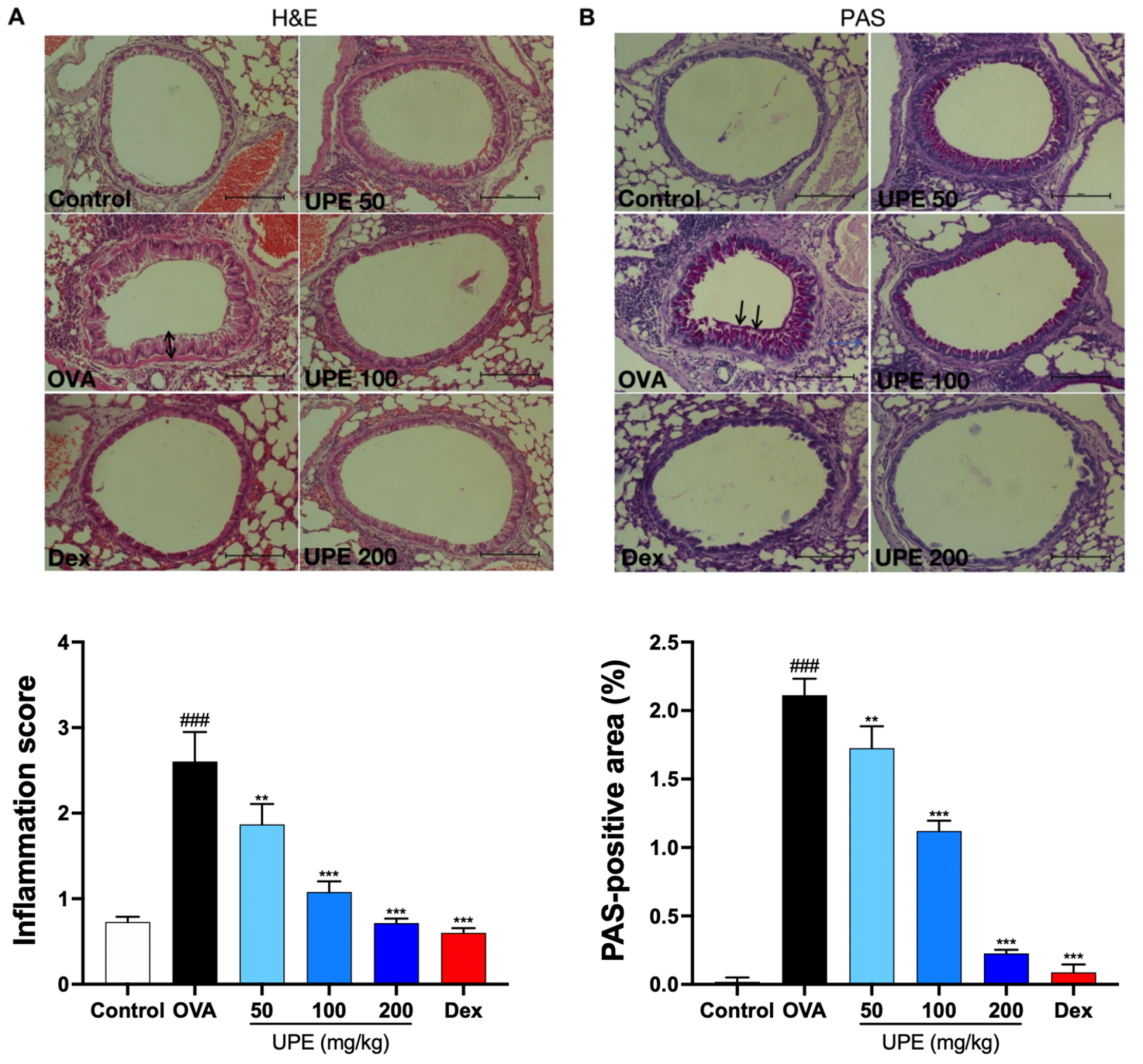
Figure 3 Alleviation of airway inflammation in lung tissues by UPE in OVA-induced CARAS mice (A) The inflammatory response in the lungs was examined by hematoxylin and eosin (H&E) staining. (B) Histological changes were examined via periodic acid-Schiff (PAS) staining. Scale bars: 100 μm. The values (n = 6) represent the means ± SEM of triplicate experiments. Data were presented as the mean ± SEM of triplicate experiments. One-way ANOVA was used to compare the differences among multiple groups. *P < 0.05, **P < 0.01, ***P < 0.001 vs OVA group; ### P < 0.001 vs control group. n = 6.

To confirm the potential effect of UPE on CARAS mice, the mice were challenged with OVA as described previously and then treated with UPE for 13 days, and typical histopathological changes were observed through H&E ( Figure 3A) and PAS ( Figure 3B) staining. Epithelial hyperplasia and mucous secretion in the lung tissue of the OVA treatment group were observed. However, UPE or Dex treatment ameliorated OVA-induced morphological changes.

### UPE alleviates nasal inflammation in OVA-induced CARAS mice

**Figure 4:**
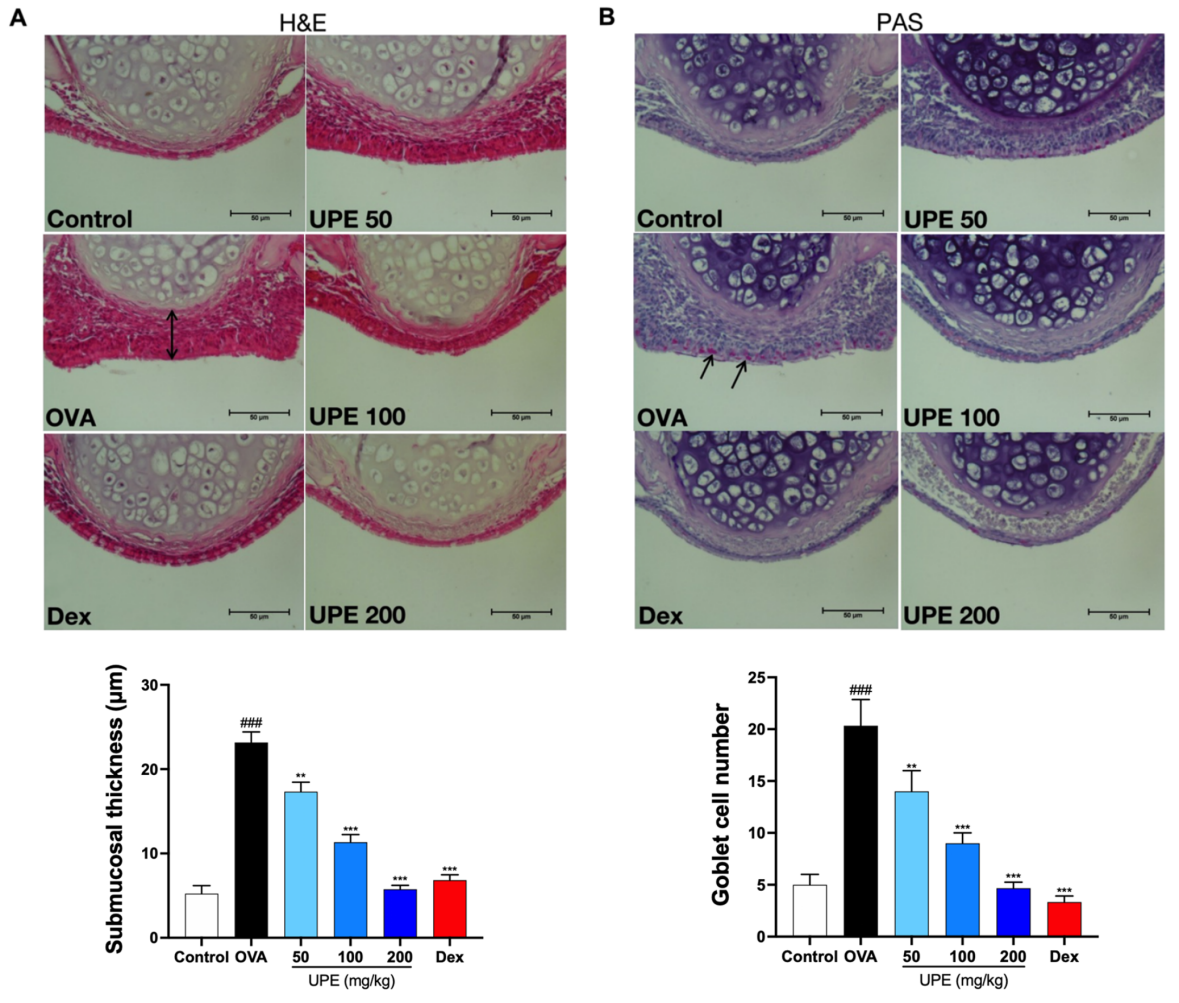
Figure 4 Alleviation of nasal inflammation in the nasal mucosa by UPE in OVA-induced CARAS mice (A) Submucosal thickness was examined by hematoxylin and eosin (H&E) staining. (B) Clobet cell numbers were examined via periodic acid-Schiff (PAS) staining. Scale bars: 50 μm. Data were presented as the mean ± SEM of triplicate experiments. One-way ANOVA was used to compare the differences among multiple groups. *P < 0.05, **P < 0.01, ***P < 0.001 vs OVA group; ### P < 0.001 vs control group. n = 6.

To determine whether UPE could suppress allergic responses in CARAS, pathological changes in the nasal mucosa were analysed. As shown in Figure 4, the nasal mucosa of the control mice presented a normal structure ( Figure 4A) and little goblet cell hyperplasia ( Figure 4B). A thicker epithelial layer and more goblet cell hyperplasia were apparent in the OVA group. However, UPE or Dex administration significantly alleviated the pathological changes in the nasal mucosa.

### UPE modulates OVA-specific Igs in the serum of OVA-induced CARAS mice

**Figure 5:**
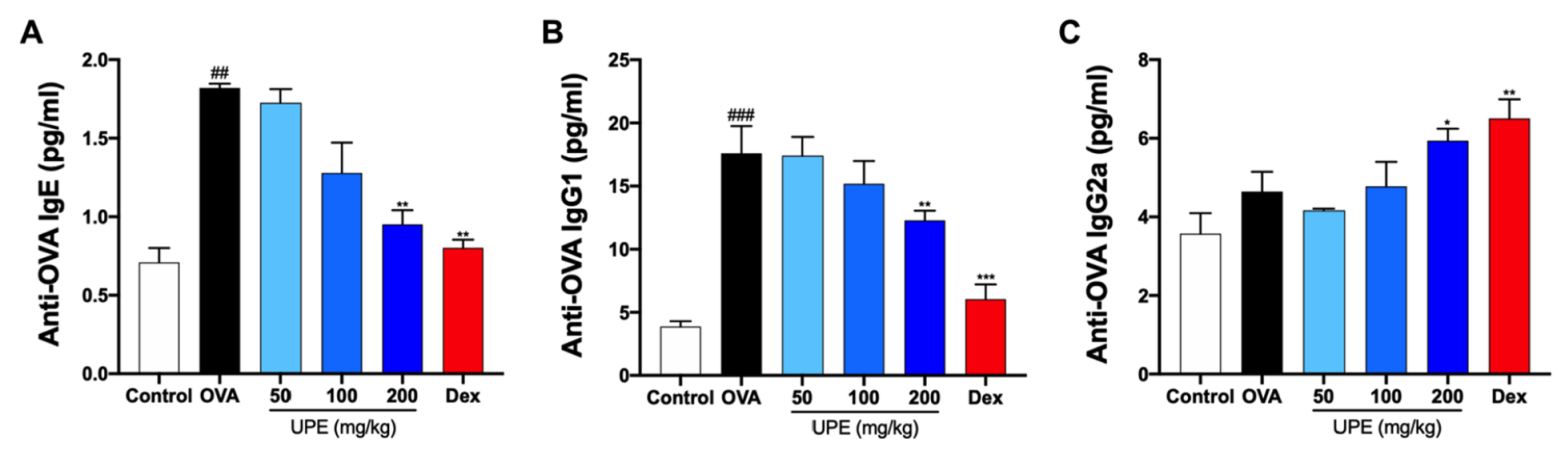
Figure 5 Reduction in OVA-specific antibody levels in the serum caused by UPE in OVA-induced CARAS mice (A) The level of OVA-specific IgE in the serum was analyzed by ELISA kit. (B) The level of OVA-specific IgG1 in the serum was analyzed by ELISA kit. (C) The level of OVA-specific IgG2a in the serum was analyzed by ELISA kit. Data were presented as the mean ± SEM of triplicate experiments. One-way ANOVA was used to compare the differences among multiple groups. *P < 0.05, ***P < 0.001 vs OVA group; ### P < 0.001 vs control group. n = 6.

It is well known that antibodies play pivotal roles in the immune system [23]. The changes in the levels of OVA-specific antibodies, including OVA-specific IgE, IgG1, and IgG2a, were quantified via an ELISA kit. Compared with those in the control group, the levels of both OVA-specific IgE and IgG1 in the OVA group were markedly increased ( Figure 5A,B). However, after UPE or Dex treatment, the levels of OVA-specific IgE and IgG1 were decreased in OVA-induced CARAS mice. In addition, the level of OVA-specific IgG2a was decreased in the OVA group and increased after UPE or Dex treatment ( Figure 5C).

### UPE regulates Th1/Th2-related cytokines in the NALF and BALF of OVA-induced CARAS mice

**Figure 6:**
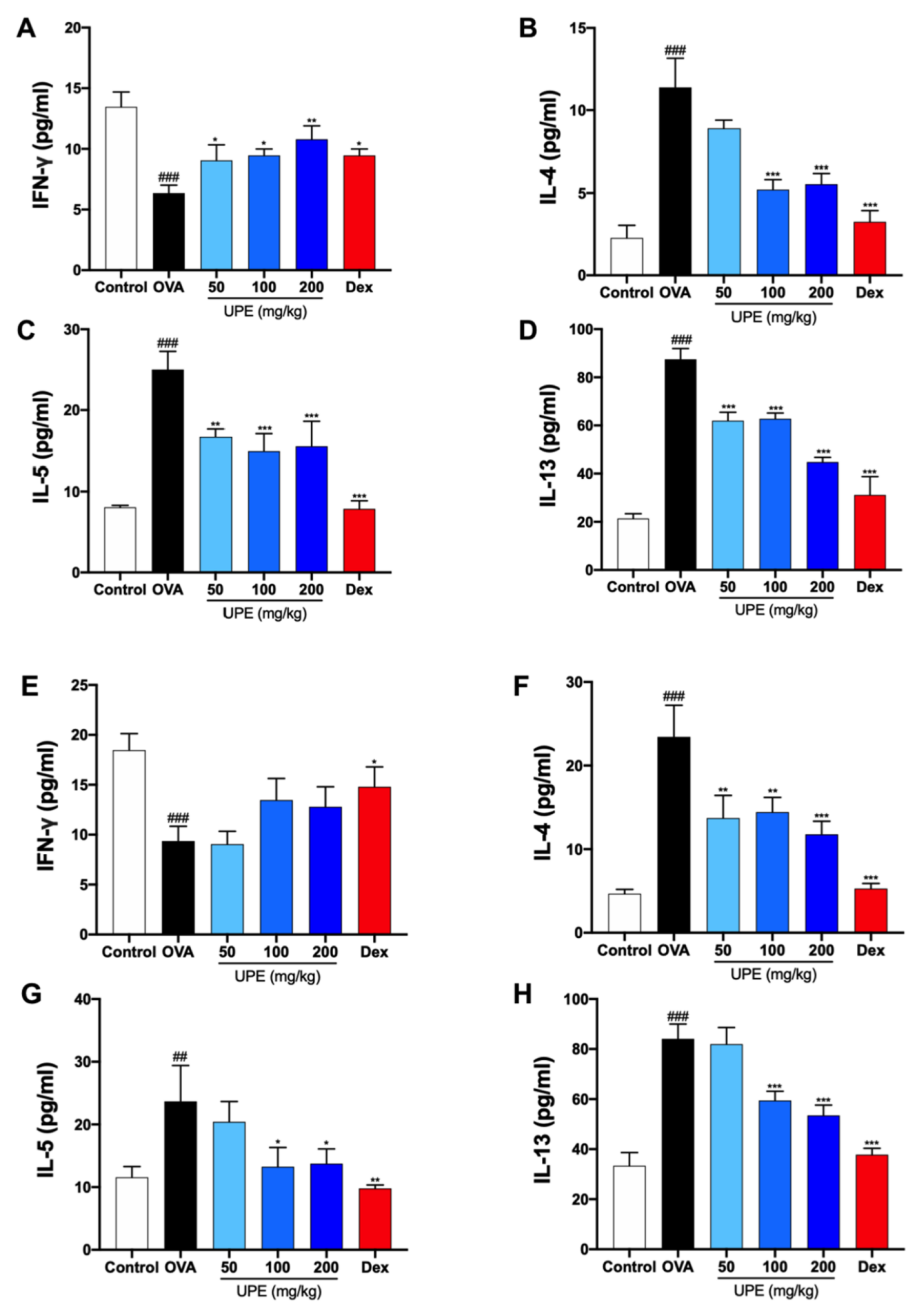
Figure 6 Regulation of Th1/Th2-related cytokines by UPE in OVA-induced CARAS mice (A–D) IFN-γ, IL-4, IL-5, and IL-13 levels in NALF were examined via ELISA kits. (E–H) IFN-γ, IL-4, IL-5, and IL-13 levels in the BALF were examined via ELISA kits. Data were presented as the mean±SEM of triplicate experiments. One-way ANOVA was used to compare the differences among multiple groups. *P < 0.05, ***P < 0.001 vs OVA group; ### P < 0.001 vs control group. n = 6.

We examined Th1 (IFN-γ) and Th2 (IL-4, IL-5, and IL-13) cytokine levels in NALF and BALF to investigate the effects of UPE on the regulation of T helper cell responses. CARAS is accompanied by a decrease in the Th1 inflammatory response. As shown in Figure 6A,E, the level of IFN-γ was significantly decreased in the OVA-treated group, whereas the level of IFN-γ was increased after UPE and Dex treatment. Th2 cells are involved in the allergic inflammatory response. Th2 (IL-4, IL-5, and IL-13) cytokines were increased during inflammatory conditions in the OVA group. However, the levels of these cytokines were notably decreased following treatment with UPE or Dex in both the NALF and BALF ( Figure 6B‒D,F‒H).

### UPE regulates the expressions of oxidative stress mediators in OVA-induced CARAS mice

**Figure 7:**
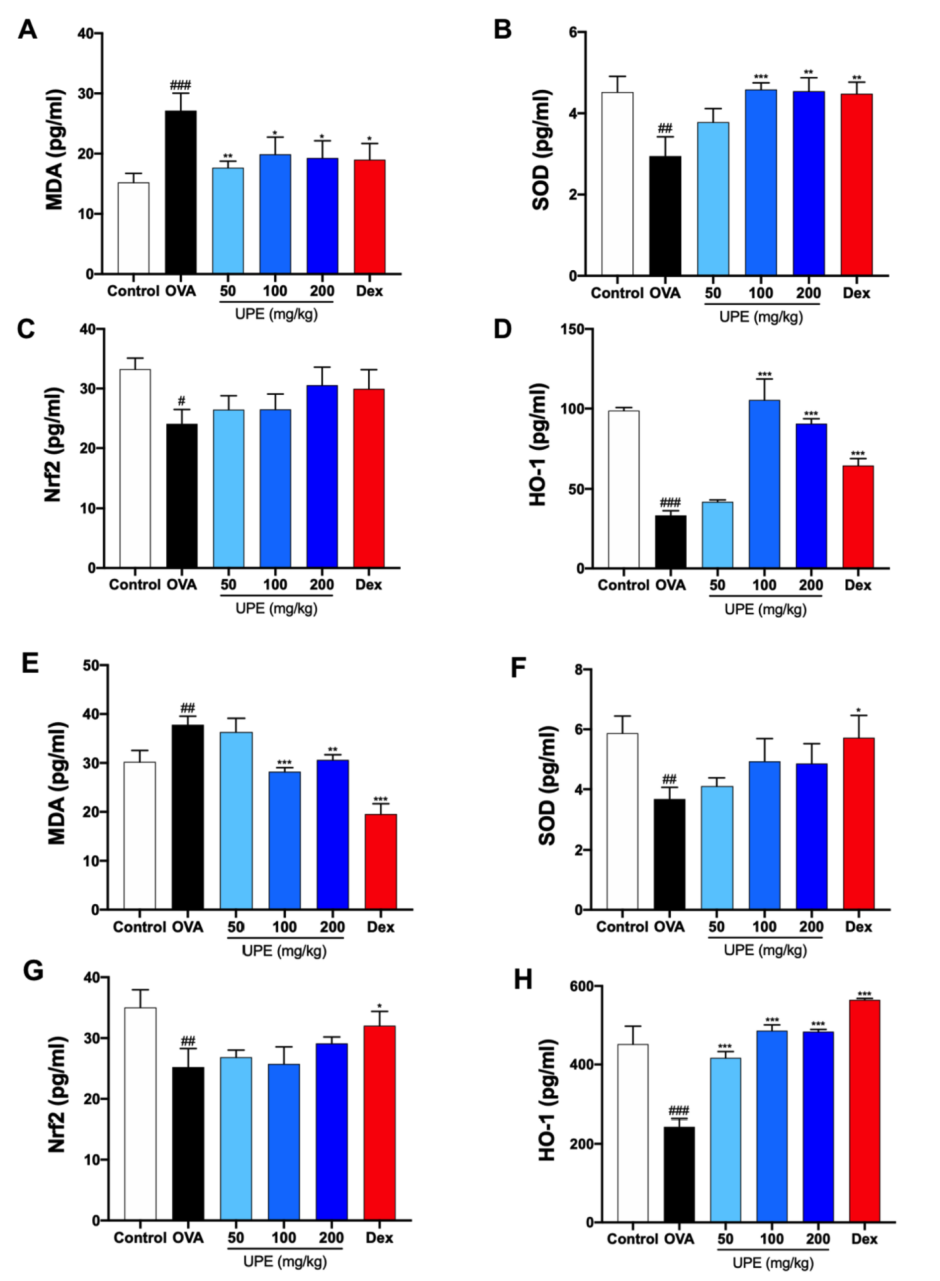
Figure 7 Modulation of oxidative stress mediator expression in NALF and BALF by UPE in OVA-induced CARAS mice (A‒D) MDA, SOD, Nrf2, and HO-1 levels in NALF were examined via ELISA kits. (E‒H) MDA, SOD, Nrf2, and HO-1 levels in the BALF were examined via ELISA kits. Data were presented as the mean ± SEM of triplicate experiments. One-way ANOVA was used to compare the differences among multiple groups. *P < 0.05, ***P < 0.001 vs OVA group; ### P < 0.001 vs control group. n = 6.

Airway oxidative stress can promote type 2 immune responses [24]. We examined the levels of antioxidant factors (SOD, Nrf2, and HO-1) and oxidative stress factor (MDA) in NALF and BALF to investigate the effects of UPE on the regulation of antioxidant function. The levels of SOD, Nrf2 and HO-1 in both the NALF and BALF decreased in the OVA group. Compared with OVA treatment, UPE or Dex treatment markedly increased the levels of SOD, Nrf2 and HO-1 in both NALF and BALF ( Figure 7B‒D,F‒H) and significantly decreased the MDA level ( Figure 7A,E).

### UPE modulates tight junction protein expression in OVA-induced CARAS mice

**Figure 8:**
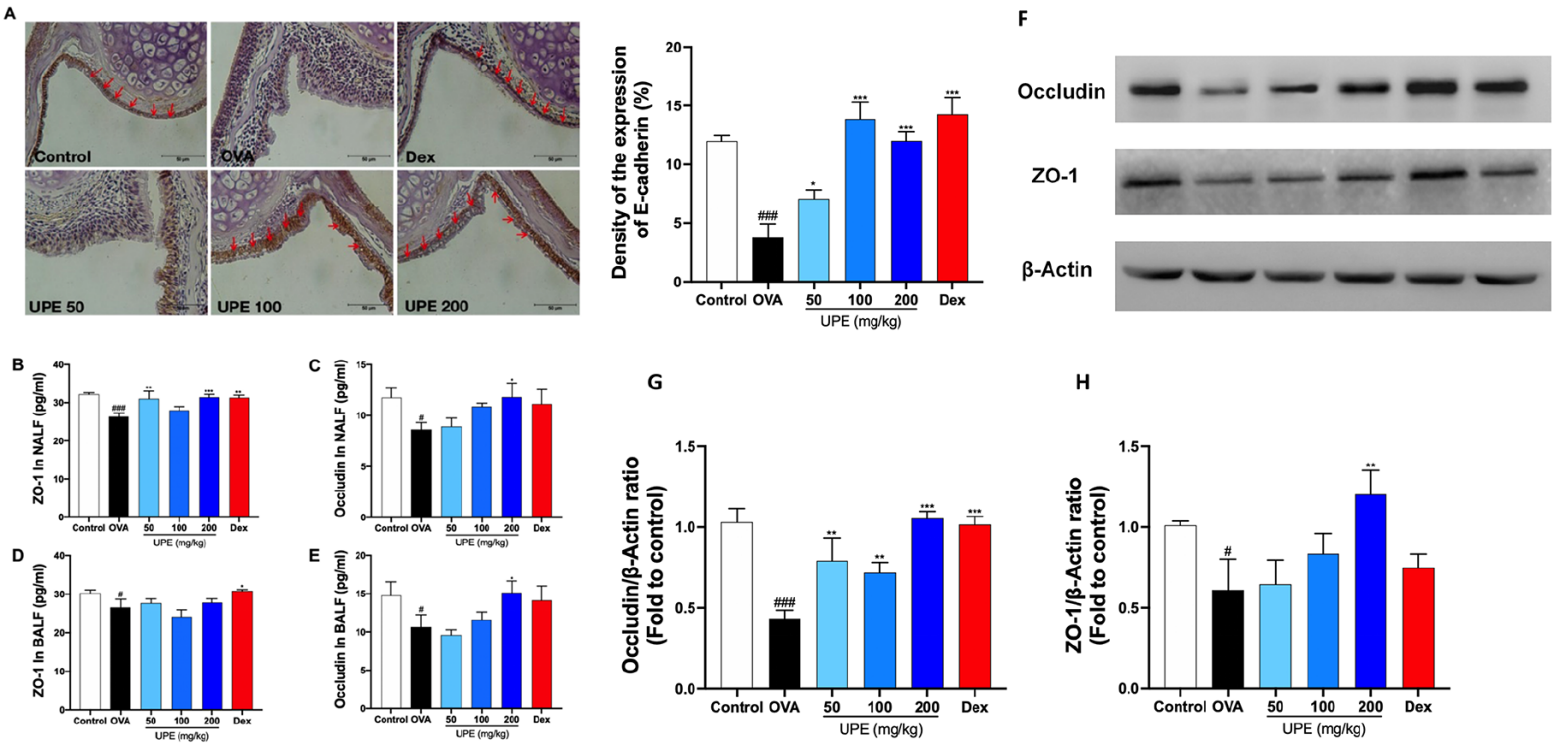
Figure 8 Regulation of tight junction protein expression by UPE in OVA-induced CARAS mice (A) Immunohistochemical labelling revealed E-cadherin expression in the epithelial layer. (B,C) ZO-1 and occludin levels in NALF were examined via ELISA kits. (D,E) ZO-1 and occludin levels in the BALF were examined by ELISA kits. (F‒H) ZO-1 and occludin protein levels in lung tissues were examined by western blot analysis. Data were presented as the mean ± SEM of triplicate experiments. One-way ANOVA was used to compare the differences among multiple groups. *P < 0.05, ***P < 0.001 vs OVA group; ### P < 0.001 vs control group. n = 6. Scale bars: 50 μm.

Oxidative stress caused by inflammation has been reported to disrupt epidermal barrier function [25]. Therefore, to investigate whether UPE repairs epithelial barrier damage in CARAS, the levels of ZO-1, Occludin and E-cadherin were measured. Immunohistochemical staining revealed that E-cadherin expression was significantly reduced in OVA-induced CARAS mice, but E-cadherin expression was significantly increased after UPE treatment ( Figure 8A). Moreover, the ELISA results suggested that the ZO-1 and occludin levels in the NALF and BALF were decreased in OVA-induced CARAS mice, whereas treatment with UPE or Dex significantly increased the ZO-1 and occludin levels ( Fig. 8B–E). Consistently, western blot analysis revealed that the protein levels of ZO-1 and Occludin were reduced in the OVA group, whereas UPE or Dex treatment increased the protein levels of ZO-1 and Occludin ( Figure 8F‒H).

### UPE inhibits the NF-κB/MAPK signaling pathway in OVA-induced CARAS mice

**Figure 9:**
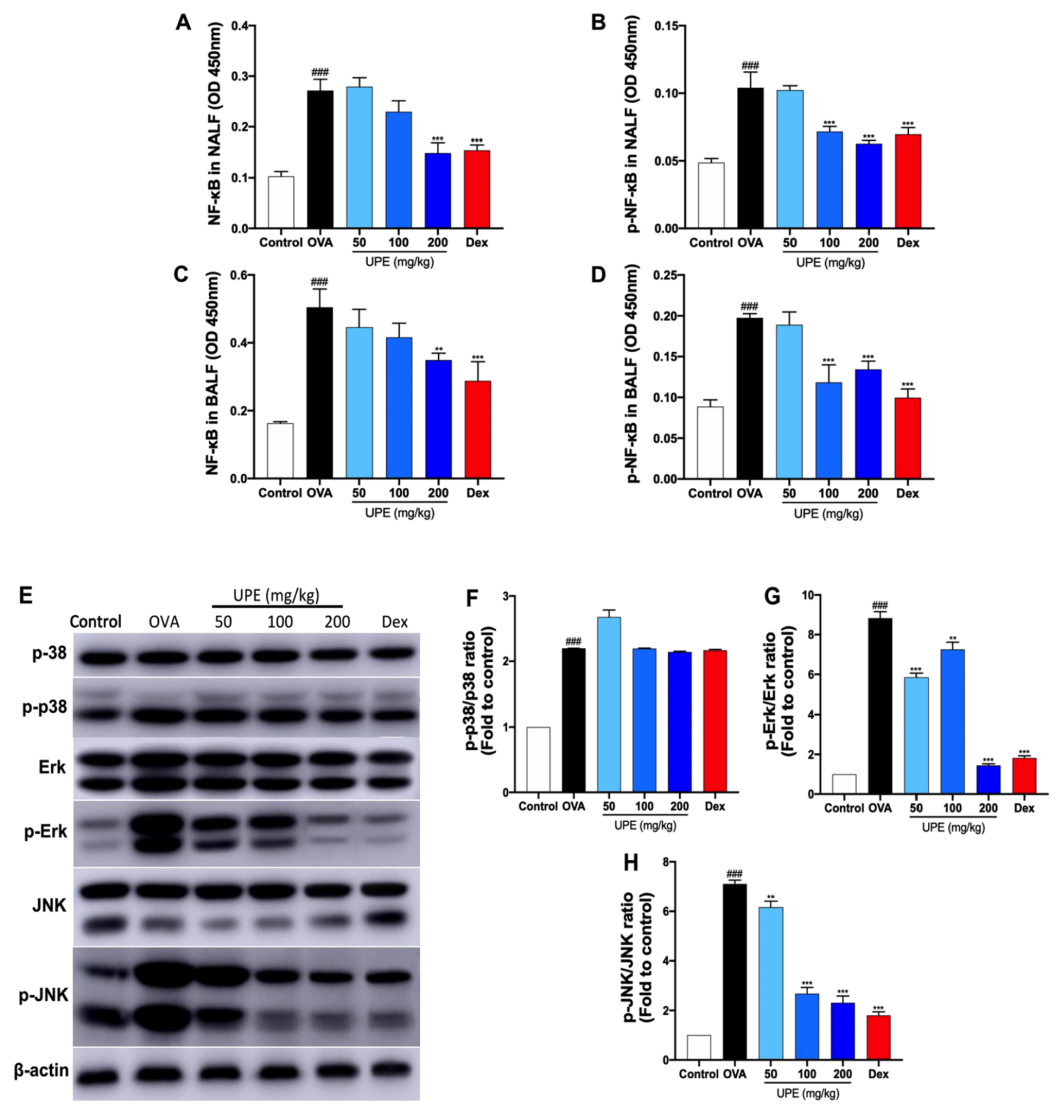
Figure 9 Inhibition of the NF-κB/MAPK signaling pathway by UPE in OVA-induced CARAS mice (A,B) NF-κB and NF-κB phosphorylation levels in NALF were examined via ELISA kits. (C,D) NF-κB and NF-κB phosphorylation levels in the BALF were examined via ELISA kits. (E) Western blot analysis of MAPKs. (F‒H) Quantification of the p-p38/p38 ratio, p-Erk/Erk ratio, and p-JNK/JNK ratio. Data were presented as the mean ± SEM of triplicate experiments. One-way ANOVA was used to compare the differences among multiple groups. *P < 0.05, ***P < 0.001 vs OVA group; ### P < 0.001 vs control group. n = 6.

NF-κB is a transcription factor that plays a crucial role in regulating the inflammatory response [26]. MAPKs, such as p38MAPK, extracellular signal-regulated kinase (Erk), and c-Jun N-terminal kinase (JNK), are identified as central regulators of inflammation [27]. As shown in Figure 9A‒H, compared with control mice, OVA-challenged mice presented significantly greater total NF-κB levels, NF-κB phosphorylation in NALF and BALF, and MAPK phosphorylation in lung tissues. However, UPE or Dex treatment significantly inhibited the phosphorylation of NF-κB and MAPKs compared with that in OVA-challenged mice.

### UPE inhibits the IL-33/ST2 level in OVA-induced CARAS mice

**Figure 10:**
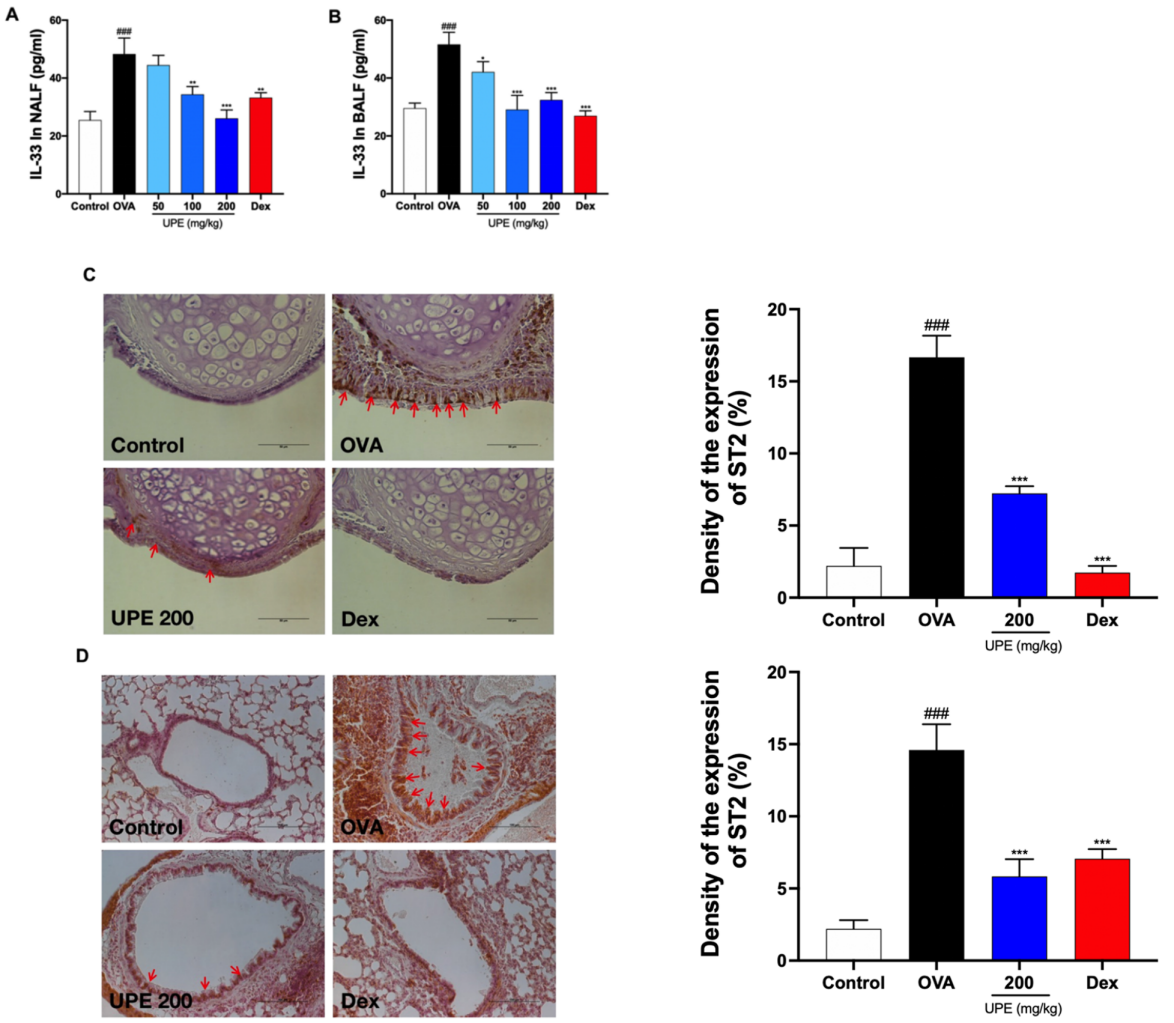
Figure 10 Inhibition of IL-33/ST2 expression by UPE in OVA-induced CARAS mice (A) IL-33 levels in the NALF in different groups were detected by an ELISA kit. (B) IL-33 in the BALF of different groups was detected with an ELISA kit. (C) ST2 in the nasal tissue was examined by immunohistochemistry. (D) ST2 in the lung was examined by immunohistochemical staining. Data were presented as the mean ± SEM of triplicate experiments. One-way ANOVA was used to compare the differences among multiple groups. *P < 0.05, ***P < 0.001 vs OVA group; ### P < 0.001 vs control group. n = 6. Scale bars: 50 or 100 μm.

IL-33-ST2, also known as the IL-1RL1 axis, is considered the chief culprit in allergic diseases such as asthma, atopic dermatitis, and autoimmune disorders [28]. Therefore, the level of IL-33 was measured with an ELISA kit. As shown in Figure 10A,B, compared with control mice, OVA-challenged mice presented significantly greater IL-33 levels in NALF and BALF. However, UPE or Dex treatment significantly decreased the IL-33 level in OVA-challenged mice. ST2, the receptor of IL-33, is distributed mainly in mouse epithelial cells and inflammatory cell membranes. Immunohistochemical staining of mouse lung and nasal tissues revealed that the level of the ST2 protein was significantly elevated in OVA-challenged mice, whereas the level of the ST2 protein was significantly reduced after UPE (200 mg/kg) or Dex treatment ( Figure 10C,D).

## Discussion

Allergic rhinitis and asthma syndromes are considered single diseases of the respiratory tract. Since they are triggered by the same properties as pathogens and present the exact characteristics of the cellular and humoral immune responses, their origin is closely related to and mediates inflammatory processes [29]. In addition, many drugs are used as antiallergic therapies that can relieve the symptoms of both diseases, and long-term treatment can produce potentially serious side effects [30]. In recent years, the application of marine algae in nutritional health/functional foods, cosmetics/cosmeceuticals and pharmaceuticals has attracted increasing attention [31]. *U*. *pinnatifida* is commonly found in the brown algae of three major marine algae, and sulfated galactans are isolated from U. pinnatifida and have anti-tumor activity [32]. In addition, the immunomodulation and mechanisms of fucoidans can improve the symptoms of atopic dermatitis [33]. These factors have important therapeutic implications and demonstrate the potential of studying new, natural, synthetic, or semi-synthetic molecules to modulate the immune response and reduce side effects in allergic patients.

In the present study, we investigated the anti-allergic and anti-inflammatory functions of UPE using an OVA-induced mouse model of CARAS. To demonstrate the effects of UPE on epithelial oxidative stress and barrier dysfunction in OVA-induced allergic reactions, we evaluated nasal symptoms, OVA-specific antibodies, infiltration of inflammatory cells into the nasal mucosa, and epithelial barrier integrity. In addition, the NF-κB/MAPK signalin pathway was analyzed to determine the anti-inflammatory effect of UPE. Consistently, our previous study explored the role of UPE in allergic rhinitis and demonstrated that UPE inhibited inflammation by regulating the NFhκF/MAPK signaling pathway and suppressing the activation of critical immune cells such as eosinophils and mast cells [22]. However, our novel study explored the effects of UPE on oxidative stress and barrier dysfunction in an OVA-induced mouse model of CARAS. In addition, our study revealed that UPE inhibited the IL-33/ST2 level in OVA-induced CARAS mice, further revealing the mechanism of action of UPE in CARAS.

In the experimental model of CARAS, mice developed an airway inflammatory response after allergen exposure, accompanied by airway remodelling and hyperreactivity. Extensive infiltration of immune cells, especially eosinophils and neutrophils, leads to oxidative stress in mice by inactivating the antioxidant enzyme SOD in membrane phospholipids, resulting in overproduction of ROS and MDA [34]. However, in a previous study, increased expression of the anti-oxidative mediator HO-1 induced by activated Nrf2 led to reduced intestinal mucosal damage and tight junction dysfunction in a rat liver transplantation model [35]. Additionally, oxidative stress causes respiratory complications of CARAS by inducing hyperresponsiveness, disassembly of the ZO-1, occludin and E-cadherin proteins, and epithelial barrier integrity [36]. IL-33 belongs to the IL-1 cytokine family and is expressed mainly during epithelial and fibroblast-like cell damage and the inflammatory response [37]. Current studies have shown that IL-33 not only induces Th0 cells to differentiate into Th2 cells but also promotes the release of the Th2 cytokines IL-4, IL-5, and IL-13 in vitro and in vivo [38]. In a study of children with asthma, the proportions of Th2 cells, eosinophils, and mast cells and the level of IL-33 in the serum were significantly increased, and there was a positive correlation with the level of the autoantibody IgE in the body [39]. Another characteristic of the experimental model of CARAS is the development of a type 2 immune response related to the Th2 cytokine profile [4]. The increase in this type of immunoglobulin is related to the atopic clinical condition of the allergic individual because it binds to a specific receptor (FcεRI) present in mast cells and eosinophils. Indeed, in these cells, the binding of the allergen to the IgE-FcεRI complex activates an intracellular cascade that ultimately leads to the degranulation process and the release of mediators that cause clinical symptoms of allergy (i.e., sneezing and rubbing) [ 40, 41] . All of these effects depend on greater activation of the transcription factor NF-ĸB and MAPKs. In addition, high levels of IL-13 in airway compartments act on goblet cells, inducing mucus production and blocking air flux [42]. Therefore, any drug that blocks these events could serve as a prototype anti-allergy molecule.

In this study, UPE treatment reduced the release of the pro-oxidant factor MDA in epithelial cells while increasing the release of the antioxidant factors SOD, Nrf2 and HO-1. The levels of tight junction-associated proteins, including E-cadherin, ZO-1, and occludin, were dramatically increased in the mice treated with UPE, which effectively limited epithelial loss. Immune staining clarified the role of UPE in protecting the integrity of E-cadherin in epithelial cells. Because UPE treatment attenuated the oxidative stress and barrier function damage induced by airway epithelial cells in mice in the presence of allergens, the release of IL-33 from epithelial cells was also inhibited. Therefore, the type 2 immune response induced by the inflammatory cytokine IL-33 was also further inhibited. We detected a reduction in the serum OVA-specific IgE and IgG1 levels in UPE-treated mice. Moreover, in the UPE group, the decrease in serum OVA-specific antibodies corresponded to a decrease in allergic symptoms, such as sneezing and rubbing scores. In this study, OVA-exposed mice had a greater number of eosinophils in both NALF and BALF. These results revealed that the bronchial and nasal mucosa layers were swollen and that inflammatory cells infiltrated extensively. Moreover, UPE inhibited airway eosinophilia and thickness in mice in a dose-dependent manner. Indeed, in mouse models, OVA challenge induced the overexpression of type 2 cytokines through the NF-κB/MAPK pathway by increasing NF-κB translocation to the cell nucleus. In this study, we demonstrated p65 NF-κB activation and p38 MAPK reduction in UPE-treated mice. This ultimately suppressed the production of type 2 cytokines during anaphylaxis. Consistent with our findings, Kim *et al*. [24] reported that Fucoidans isolated from the sporophylls of Undaria pinnatifida effectively alleviate particulate matter-exacerbated allergic asthma symptoms by attenuating the airway inflammatory response, oxidative stress and mucus hypersecretion. Since UPE is a plant extract, further preclinical studies and better animal models are needed to close the knowledge gap between animal and human studies. The current study provides a thorough understanding of the mechanism of action, which will accelerate the development of clinical trial protocols for UPE soon.

In conclusion, we demonstrated that UPE inhibited IL-33 release caused by epithelial cell destruction by attenuating oxidative stress and dysfunction in mouse epithelial cells. UPE treatment also reduced the entry of eosinophils into the upper and lower airway lumen and modulated the type 2 immune response transcription factor pathway. Moreover, UPE inhibited OVA-mediated inflammatory processes in both airway compartments of CARAS mice. These findings revealed that UPE might be a viable immunotherapy approach for lung diseases such as CARAS. Patients can choose the appropriate dose of UPE according to the severity of CARAS.

## Supporting information

24502Supplementary_Figures

## Supplementary Data

Supplementary data is available at *Acta Biochimica et Biophysica Sinica* online.

## References

[1] Paiva Ferreira LKD, Paiva Ferreira LAM, Monteiro TM, Bezerra GC, Bernardo LR, Piuvezam MR (2019). Combined allergic rhinitis and asthma syndrome (CARAS). Int Immunopharmacol.

[2] Leynaert B, Neukirch F, Demoly P, Bousquet J (2000). Epidemiologic evidence for asthma and rhinitis comorbidity. J Allergy Clin Immunol.

[3] Taramarcaz P, Gibson PG (2004). The effectiveness of intranasal corticosteroids in combined allergic rhinitis and asthma syndrome. Clin Exp Allergy.

[4] Cavalcanti RFP, Gadelha F, de Jesus TG, Cavalcante-Silva LHA, Paiva Ferreira LKD, Paiva Ferreira LAM, Vieira GC (2020). Warifteine and methylwarifteine inhibited the type 2 immune response on combined allergic rhinitis and asthma syndrome (CARAS) experimental model through NF-кB pathway. Int Immunopharmacol.

[5] Wang X, Liu C, Wu L, Zhu S (2016). Potent ameliorating effect of Hypoxia-inducible factor 1α (HIF-1α) antagonist YC-1 on combined allergic rhinitis and asthma syndrome (CARAS) in rats. Eur J Pharmacol.

[6] Nakahara T, Moroi Y, Uchi H, Furue M (2006). Differential role of MAPK signaling in human dendritic cell maturation and Th1/Th2 engagement. J Dermatological Sci.

[7] Nesi RT, Barroso MV, Souza Muniz V, de Arantes AC, Martins MA, Brito Gitirana L, Neves JS (2017). Pharmacological modulation of reactive oxygen species (ROS) improves the airway hyperresponsiveness by shifting the Th1 response in allergic inflammation induced by ovalbumin. Free Radical Res.

[8] Buckley A, Turner JR (2018). Cell biology of tight junction barrier regulation and mucosal disease. Cold Spring Harb Perspect Biol.

[9] Hammad H, Lambrecht BN (2015). Barrier epithelial cells and the control of type 2 immunity. Immunity.

[10] Holgate ST (2011). The sentinel role of the airway epithelium in asthma pathogenesis. Immunol Rev.

[11] Schiering C, Krausgruber T, Chomka A, Fröhlich A, Adelmann K, Wohlfert EA, Pott J (2014). The alarmin IL-33 promotes regulatory T-cell function in the intestine. Nature.

[12] Mitchell PD, Salter BM, Oliveria JP, El-Gammal A, Tworek D, Smith SG, Sehmi R (2018). IL-33 and its receptor ST2 after inhaled allergen challenge in allergic asthmatics. Int Arch Allergy Immunol.

[13] Montero L, Herrero M, Ibáñez E, Cifuentes A (2014). Separation and characterization of phlorotannins from brown algae
*Cystoseira abies‐marina* by comprehensive two‐dimensional liquid chromatography. Electrophoresis.

[14] Wang T, Jónsdóttir R, Liu H, Gu L, Kristinsson HG, Raghavan S, Ólafsdóttir G (2012). Antioxidant capacities of phlorotannins extracted from the brown algae fucus vesiculosus. J Agric Food Chem.

[15] Pinto E, Hrimpeng K, Lopes G, Vaz S, Gonçalves MJ, Cavaleiro C, Salgueiro L (2013). Antifungal activity of Ferulago capillaris essential oil against candida, cryptococcus, aspergillus and dermatophyte species. Eur J Clin Microbiol Infect Dis.

[16] Lee SH, Jeon YJ (2013). Anti-diabetic effects of brown algae derived phlorotannins, marine polyphenols through diverse mechanisms. Fitoterapia.

[17] Cemek B, GÜLer M, KiliÇ K, Demir Y, Arslan H (2007). Assessment of spatial variability in some soil properties as related to soil salinity and alkalinity in Bafra plain in northern Turkey. Environ Monit Assess.

[18] Maruyama H, Tamauchi H, Hashimoto M, Nakano T (2005). Suppression of Th2 immune responses by mekabu fucoidan from undaria pinnatifida sporophylls. Int Arch Allergy Immunol.

[19] Zayed A, Avila-Peltroche J, El-Aasr M, Ulber R (2022). Sulfated galactofucans: an outstanding class of fucoidans with promising bioactivities. Mar Drugs.

[20] Lee JB, Hayashi K, Hashimoto M, Nakano T, Hayashi T (2004). Novel antiviral fucoidan from sporophyll of undaria pinnatifida (mekabu). Chem Pharm Bull.

[21] Agnihotri NT, McGrath KG (2019). Allergic and nonallergic rhinitis. allergy asthma proc.

[22] Yu ZN, Fan YJ, Nguyen TV, Piao CH, Lee BH, Lee SY, Shin HS (2024). Undaria pinnatifida ameliorates nasal inflammation by inhibiting eosinophil and mast cell activation and modulating the NF‐κB/MAPKs signaling pathway. Immun Inflam Dis.

[23] Yanase N, Toyota H, Hata K, Yagyu S, Seki T, Harada M, Kato Y (2014). OVA-bound nanoparticles induce OVA-specific IgG1, IgG2a, and IgG2b responses with low IgE synthesis. Vaccine.

[24] Herath KHINM, Kim HJ, Kim A, Sook CE, Lee BY, Jee Y (2020). The role of fucoidans isolated from the sporophylls of
*Undaria pinnatifida* against particulate-matter-induced allergic airway inflammation: evidence of the attenuation of oxidative stress and inflammatory responses. Molecules.

[25] Chen Y, Li X, Gan X, Qi J, Che B, Tai M, Gao S (2019). Fucoidan from
*Undaria pinnatifida* ameliorates epidermal barrier disruption via keratinocyte differentiation and CaSR level regulation. Mar Drugs.

[26] Zhang Q, Wang L, Chen B, Zhuo Q, Bao C, Lin L (2017). Propofol inhibits NF-κB activation to ameliorate airway inflammation in ovalbumin (OVA)-induced allergic asthma mice. Int Immunopharmacol.

[27] Kim MG, Kim SM, Min JH, Kwon OK, Park MH, Park JW, Ahn HI (2019). Anti-inflammatory effects of linalool on ovalbumin-induced pulmonary inflammation. Int Immunopharmacol.

[28] Takatori H, Makita S, Ito T, Matsuki A, Nakajima H (2018). Regulatory mechanisms of IL-33-ST2-Mediated allergic inflammation. Front Immunol.

[29] Liu X, Pan B, Sun L, Chen X, Zeng K, Hu X, Xu T (2018). Circulating exosomal miR-27a and miR-130a act as novel diagnostic and prognostic biomarkers of colorectal cancer. Cancer Epidemiol Biomarkers Prevention.

[30] Meltzer EO, Blaiss MS, Naclerio RM, Stoloff SW, Derebery MJ, Nelson HS, Boyle JM (2012). Burden of allergic rhinitis: allergies in america, latin america, and Asia-Pacific adult surveys. allergy asthma proc.

[31] Ngo DH, Kim SK (2013). Sulfated polysaccharides as bioactive agents from marine algae. Int J Biol Macromol.

[32] Vishchuk OS, Ermakova SP, Zvyagintseva TN (2011). Sulfated polysaccharides from brown seaweeds
*Saccharina japonica* and
*Undaria pinnatifida*: isolation, structural characteristics, and antitumor activity. Carbohydrate Res.

[33] Chen BR, Hsu KT, Hsu WH, Lee BH, Li TL, Chan YL, Wu CJ (2021). Immunomodulation and mechanisms of fucoidan from cladosiphon okamuranus ameliorates atopic dermatitis symptoms. Int J Biol Macromol.

[34] Xu C, Song Y, Wang Z, Jiang J, Piao Y, Li L, Jin S (2021). Pterostilbene suppresses oxidative stress and allergic airway inflammation through AMPK/Sirt1 and Nrf2/HO‐1 pathways. Immun Inflam Dis.

[35] Ahmed SM, Luo L, Namani A, Wang XJ, Tang X (2017). Nrf2 signaling pathway: pivotal roles in inflammation. Biochim Biophys Acta Mol Basis Dis.

[36] Yamamoto N, Kan-o K, Tatsuta M, Ishii Y, Ogawa T, Shinozaki S, Fukuyama S (2021). Incense smoke-induced oxidative stress disrupts tight junctions and bronchial epithelial barrier integrity and induces airway hyperresponsiveness in mouse lungs. Sci Rep.

[37] Nian JB, Zeng M, Zheng J, Zeng LY, Fu Z, Huang QJ, Wei X (2020). Epithelial cells expressed IL-33 to promote degranulation of mast cells through inhibition on ST2/PI3K/mTOR-mediated autophagy in allergic rhinitis. Cell Cycle.

[38] Finlay CM, Stefanska AM, Walsh KP, Kelly PJ, Boon L, Lavelle EC, Walsh PT (2016). Helminth products protect against autoimmunity via innate type 2 cytokines IL-5 and IL-33, which promote eosinophilia. J Immunol.

[39] Han X, Chai R, Qi F, Bai S, Cui Y, Teng Y, Liu B (2017). Natural helper cells mediate respiratory syncytial virus-induced airway inflammation by producing type 2 cytokines in an IL-33-dependent manner. Immunotherapy.

[40] Gould HJ, Sutton BJ (2008). IgE in allergy and asthma today. Nat Rev Immunol.

[41] Lambrecht BN, Hammad H (2015). The immunology of asthma. Nat Immunol.

[42] Gour N, Wills-Karp M (2015). IL-4 and IL-13 signaling in allergic airway disease. Cytokine.

