# Supplementary material for: Undaria pinnatifida extract attenuates combined allergic rhinitis and asthma syndrome by the modulation of epithelial cell dysfunction and oxidative stress : Undaria pinnatifida attenuates CARAS
Source: Acta Biochim Biophys Sin (Shanghai). 2024 Dec 24;57(5):792–804. doi: 10.3724/abbs.2024190 (PMC12130702; doi:10.3724/abbs.2024190)
Supplement: 24502Supplementary_Figures [file 24502Supplementary_Figures.docx]

**
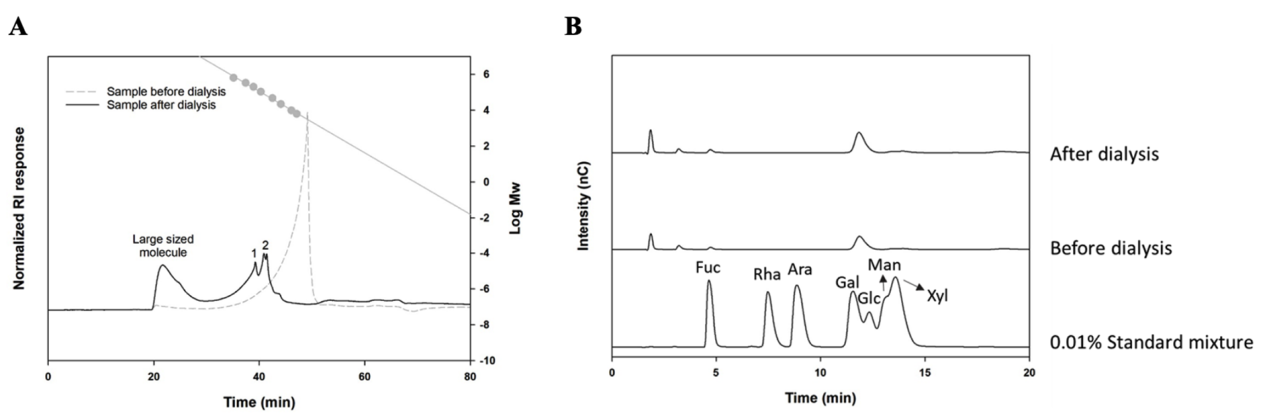
**

**Supplementary Figure S1. UPE characterization** (A) The predialysis sample included more molecules smaller than the reference material (6,300 Da), which were eliminated following dialysis. The after-dialysis sample revealed the presence of very large molecular weight chemicals (in the 20-minute range) as well as molecules mostly between 63,000‒160,000 Da (in the 1‒2 peak region). (B) The predominant sugars found in both pre- and postdialysis samples were galactose and a tiny amount of fucose, with no significant differences between the types of sugars present before and after dialysis. The amount of galactose increased following dialysis and acid hydrolysis.

**
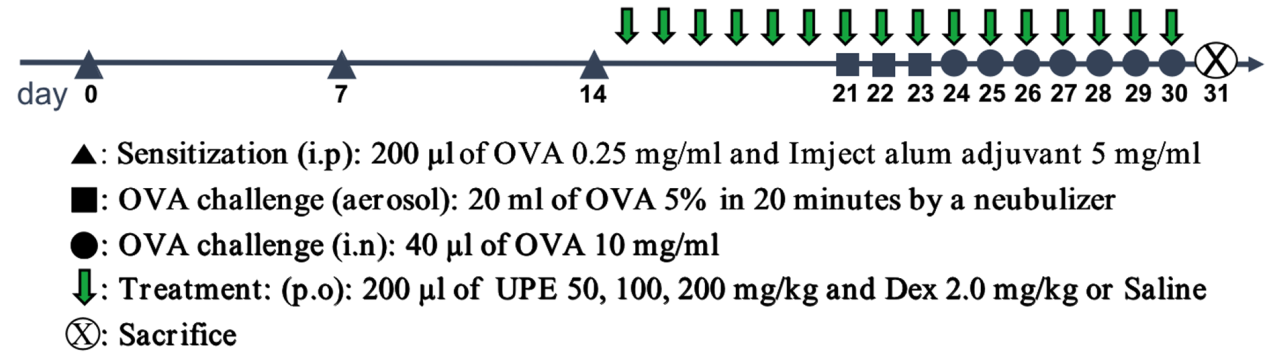
**

**Supplementary Figure S2. Experimental protocol for the establishment of combined allergic rhinitis and asthma syndrome and UPE treatment** BALB/c mice were randomly assigned to six groups: the control, OVA, UPE 50, 100, 200 mg/kg, and Dex 2 mg/kg treatment groups. The mice were sensitized on days 7 and 14. On days 21 to 23, the OVA, Dex, and UPE groups were challenged with nebulized 5% OVA solution. On days 24 to 30, the mice received an intranasal challenge in each nasal cavity with 20 μl of OVA solution. The control group received saline alone. The mice were treated with UPE and Dex orally once daily from days 15--30. The mice were sacrificed on day 31.
